# *In Vivo* Neutralization of α-Cobratoxin with High-Affinity Llama Single-Domain Antibodies (V_H_Hs) and a V_H_H-Fc Antibody

**DOI:** 10.1371/journal.pone.0069495

**Published:** 2013-07-22

**Authors:** Gabrielle Richard, Ashley J. Meyers, Michael D. McLean, Mehdi Arbabi-Ghahroudi, Roger MacKenzie, J. Christopher Hall

**Affiliations:** 1 School of Environmental Sciences, University of Guelph, Guelph, Ontario, Canada; 2 Human Health Therapeutics Portfolio, National Research Council Canada, Ottawa, Ontario, Canada; Instituto Butantan, Brazil

## Abstract

Small recombinant antibody fragments (e.g. scFvs and V_H_Hs), which are highly tissue permeable, are being investigated for antivenom production as conventional antivenoms consisting of IgG or F(ab’)_2_ antibody fragments do not effectively neutralize venom toxins located in deep tissues. However, antivenoms composed entirely of small antibody fragments may have poor therapeutic efficacy due to their short serum half-lives. To increase serum persistence and maintain tissue penetration, we prepared low and high molecular mass antivenom antibodies. Four llama V_H_Hs were isolated from an immune V_H_H-displayed phage library and were shown to have high affinity, in the low nM range, for α-cobratoxin (α–Cbtx), the most lethal component of *Naja kaouthia* venom. Subsequently, our highest affinity V_H_H (C2) was fused to a human Fc fragment to create a V_H_H2-Fc antibody that would offer prolonged serum persistence. After *in planta* (*Nicotiana benthamiana*) expression and purification, we show that our V_H_H2-Fc antibody retained high affinity binding to α–Cbtx. Mouse α–Cbtx challenge studies showed that our highest affinity V_H_Hs (C2 and C20) and the V_H_H2-Fc antibody effectively neutralized lethality induced by α–Cbtx at an antibody:toxin molar ratio as low as ca. 0.75×:1. Further research towards the development of an antivenom therapeutic involving these anti-α-Cbtx V_H_Hs and V_H_H2-Fc antibody molecules should involve testing them as a combination, to determine whether they maintain tissue penetration capability and low immunogenicity, and whether they exhibit improved serum persistence and therapeutic efficacy.

## Introduction

Snake bite envenomation is a serious global public health problem especially in tropical and subtropical countries where poisonous snakes are abundant and agriculture activities are high [1]. It is estimated that over 5 million snake bite cases occur worldwide each year, of which 2.7 million cause envenomation, with about 125,000 of these resulting in death [2]. Although many people survive envenomation, a large number of victims develop severe local tissue damage that may lead to permanent physical disability [3]. Since most snakebite victims are young male agricultural workers with families, their disability has serious social and economical impacts, especially in developing countries [1], [3].

Since their first development and use in the late 1800s by Calmette, conventional antivenoms remain the only specific treatments for envenomation. Most antivenoms are composed of either whole IgGs (150 kDa), F(ab′)_2_ antibody fragments (100 kDa) or, in some cases, Fab antibody fragments (50 kDa) from horses or sheep immunized with one (monospecific) or a mixture (polyspecific) of venoms [4]. Intravenous administration of antivenom is generally efficacious in treating systemic envenomation [3]; however, because of the rapid development of localized pathologies and the inability of antivenom antibodies to penetrate affected tissues, conventional antivenoms are generally ineffective in treating local effects on tissues near the snake bite, often resulting in permanent physical disability [3], [4], [5]. Furthermore, conventional antivenoms often elicit life-threatening adverse reactions such as anaphylaxis or serum sickness in patients [4].

Because of their unique immune system, there has been a growing interest in recent years to assess the use of camelids (camels, llamas, alpacas) for antivenom production [6], [7], [8], [9], [10], [11], [12], [13]. Aside from conventional IgGs, camelids have evolved a unique class of IgG naturally devoid of light chains called heavy-chain antibodies (HCAbs) [14]. The antigen binding sites of these HCAbs are composed of a single variable domain (called V_H_H), and are the smallest natural antigen binding domain (∼15 kDa). HCAbs, and their V_H_Hs, show particular promise for antivenom development because they can act as potent enzyme inhibitors by targeting cryptic enzyme clefts that are often poorly antigenic to mammalian immune systems that develop only conventional IgGs [15], [16]. Recent studies have shown that both camels and llamas immunized with snake venom generate antisera titres comparable to those of horses that are used for antivenom preparations [8], [17]. Encouraging results further show that camelid antibodies (unfractionated conventional IgGs and HCAbs) effectively neutralize the lethality and/or a variety of pathologies induced by snake venoms from *Bothrops mattogrossensis* (Mato Grosso lancehead) [6]; *Echis ocellathus* (saw-scaled viper) [8], [18]; and *Bitis arietans* (the puff adder) [18]. Further, scorpion (*Androctonus australis hector*) venom toxins have been shown to be potently neutralized by specific camelid IgGs, HCAbs, and by monovalent or bivalent V_H_Hs [9], [10], [11], [13]. While elapid (e.g. cobra) venoms are generally considered poorly antigenic [19], one recent study showed potent camel antisera titres in response to *Naja nigricollis* (spitting cobra) immunization [17]. Unfortunately, these camelid IgGs were not able to neutralize the lethal-effects of *N. nigricollis* envenomation in an *in vivo* mouse study [18]. This last report indicates that more research needs to be done to increase our understanding of how to treat snake envenomation, particularly by elapids, using antibody-based products.

Since conventional antivenom antibodies poorly neutralize venom toxins located in deep tissues, smaller recombinant antibody fragments (e.g. scFvs, V_H_Hs), which are highly tissue permeable, have been explored in recent years for use in antivenom preparations [9], [10], [11], [12], [20], [21], [22], [23]. In fact, camelid V_H_Hs have several attractive properties that may make them better therapeutic reagents for the treatment of snake envenomation: they are relatively non-immunogenic, soluble, stable (pH and heat), highly tissue penetrable and are easy to genetically manipulate for creation of multivalent/multifunctional formats [24], [25], [26], [27], [28], [29]. Nevertheless, antivenoms composed entirely of small antibody fragments would likely have limited therapeutic efficacy because these fragments are cleared from the body rapidly [30], [31]. Therefore, it has been suggested in recent years that antivenoms prepared with a mixture of high molecular mass antibodies (IgG; F(ab’)_2_) and low molecular mass antibody fragments (Fab; scFv; V_H_H) may offer better treatment for envenomation [8], [12], [30], [32]. This type of antivenom would not only allow the rapid neutralization of toxins by small fragments in tissue compartments, but also ensure that significant concentrations of antibodies (IgG, F(ab’)_2_) remain in circulation long enough to neutralize toxins there later in the course of envenomation.

To our knowledge, only two naïve recombinant antibody libraries have previously been panned against α-cobratoxin (α–Cbtx), the most potent α–neurotoxin from the venom of *N. kaouthia* (common names: monocellate cobra, Thai cobra). The first report was from our group, in which the isolation of three naïve llama V_H_Hs with moderate affinities, in the low µM (2–3 µM) range, to α–Cbtx was described [12]; the affinities of these were deemed too low for therapeutic efficacy and therefore were not used for *in vivo* testing. More recently, Kulkeaw et al. [21] isolated seven naïve human scFv (HuScFv) clones to α–Cbtx (affinities not reported). Their best neutralizing HuScFv (clone #24), administered at 10× Ab:toxin (w/w) (2.65 µg HuScFv:0.265 µg α–Cbtx), was only able to protect 33% of mice from α–Cbtx-induced lethality (Table 8 of [21]). Full protection against α–Cbtx was not attained even when this clone was administered at a much higher dose (83.9 µg HuScFv: 0.265 µg α–Cbtx). In this study, we set out to isolate higher affinity antibody fragments with more potent neutralizing capacity against α–Cbtx, through construction of a V_H_H library from a llama immunized with crude *N. kaouthia* venom. We report the isolation of high affinity V_H_Hs (low nM range) that offer full protection (100% mice survival) against α–Cbtx. To increase the half-life of our V_H_Hs, we also report the fusion of our highest affinity V_H_H (C2) with the Fc fragment of human IgG1 to create a V_H_H2-Fc antibody (Mr ∼80 kDa). After *in planta* expression and purification, we show that our V_H_H2-Fc antibody retained high affinity binding to α–Cbtx and also has potent *in vivo* neutralizing capacity against α–Cbtx.

## Materials and Methods

### Ethics Statement

All animal work was undertaken in strict accordance with the recommendations in the Guidelines for the Care and Use of Laboratory Animals [Canadian Council on Animal Care (CCAC), Ottawa, ON, Canada]. The protocols were approved by the Animal Care Committee of the University of Guelph (Permit Numbers: AUP-06R089 and AUP-10R009). A one-year-old male llama (*Lama glama*) was housed at a private farm (Freelton, ON, Canada) approved for animal studies by the CCAC and used for immunization and blood collection. Six to eight-week-old female ICR mice (weighing 20–30 g) were purchased from Harlan Laboratories (Indianapolis, IN, USA) and maintained at the central animal facility at the University of Guelph. For the *in vivo* neutralization assays, early humane endpoints were followed to minimize mice suffering as detailed in the ‘*In vivo* neutralization of α–Cbtx-induced lethality’ section of Materials and Methods.

### Snake Venom and α–Cbtx

*N. kaouthia* venom was purchased from Accurate Chemical & Scientific Corporation (Westbury, NY, USA), while purified α–Cbtx, derived from *N. kaouthia* venom, was purchased from Latoxan (Valence, France), both in lyophilized form. Stock solutions were prepared by reconstituting the venom or toxin in sterile phosphate buffered saline (PBS), pH 7.4 at 1 mg mL^−1^. Due to their toxicities, *N. kaouthia* venom and α–Cbtx require appropriate handling precautions, which were followed in accordance with guidelines set by the Environmental Health and Safety Department of the University of Guelph.

### Llama Immunization

Cobra venoms, and especially that of *N. kaouthia*, are believed to be weakly immunogenic because of the low molecular weight of their neurotoxins [19], [33], [34]. Therefore, we used, with our own modifications, a ‘low dose, low volume, multi-site’ venom-immunization protocol that has been shown to be effective at raising high IgG titres against cobra venoms in horses [33] and recently, in camels [17]. The llama was immunized with crude *N. kaouthia* venom and details regarding the amount, timing and adjuvants used are described in Table 1. The immunogen cocktail [0.5 mL; in phosphate buffered saline (PBS), pH 7.4] was emulsified in an equal volume of TiterMax™ Classic adjuvant (Sigma, Oakville, ON, Canada) for the first three immunizations, and with an equal volume of incomplete Freund’s adjuvant (Sigma) for subsequent immunizations. Each administration occurred subcutaneously at four different locations (0.25 mL/site) (i.e., two sites near the neck and two in the hind quarters). Llama blood was withdrawn from the jugular vein one week after immunizations 2 through 6 (Table 1). After blood coagulation, serum was obtained by centrifuging at 2,700×g for 10 min at 4°C and stored at −20°C until required. On post-immune Day 110, blood was collected for immediate isolation of peripheral blood leukocytes as described in the QIAamp RNA Blood Mini Kit (Qiagen), which were stored at −80°C until required for RNA isolation.

**Table 1:** Llama immunization schedule.

Day	Immunization number	Serum collection	Venom dose (mg)	Adjuvant
−7		Yes		
0	1		0.25	TiterMax™
14	2		0.5	TiterMax™
21		Yes		
35	3		0.75	TiterMax™
42		Yes		
56	4		1.0	IFA^a^
63		Yes		
75	5		1.25	IFA
82		Yes		
103	6		1.5	IFA
110		Yes		
127	7		2.0	IFA
134		Yes		

a IFA: incomplete Freund’s adjuvant.

The llama was immunized with crude venom of *N. kaouthia* following a low dose, low volume, multi-site protocol (see text for more details). Blood was collected from the jugular vein on the days indicated.

### Humoral Response

The llama humoral immune response to *N. kaouthia* venom and α–Cbtx were monitored over the course of the immunization schedule by indirect ELISA. Wells of a Reacti-Bind™ maleic anhydride-activated polystyrene microtitre plate (Pierce Biotechnology, Rockford, IL) were coated with either *N. kaouthia* venom or α–Cbtx (2.5 µg mL^−1^; 100 µL/well; PBS, pH 7.4) at 4°C overnight (o/n). Wells were washed 3× with 200 µL of PBS (pH 7.4) to remove unbound antigen and then blocked o/n with 300 µL of 4% M-PBS [4% (w/v) milk powder in PBS, pH 7.4]. Negative background control wells were not coated with the antigen (only blocked). Pre-immune (Day -7) and post-immune (Days 21, 42, 63, 82, 110, 134) sera were diluted 1∶25,000 in blocking buffer (2% M-PBS), added to the wells (100 µL/well), and incubated with gentle shaking at room temperature (RT). After 1.5 hr incubation, serum samples were removed and the wells were washed 3× with 200 µL of PBS-T [PBS plus 0.05% (v/v) Tween-20]. Goat anti-llama IgG-heavy and light-chain conjugated to HRP (horseradish peroxidase) (Bethyl Lab Inc, 6 Montgomery, CA, USA) diluted 1∶2,000 in 2% M-PBS was added to the wells (100 µL/well) and incubated for 1 hr at RT with gentle shaking. Wells were washed 3× with 200 µL of PBS-T, and then developed with 100 µL/well of TMB substrate (3,3′,5,5′-tetramethyl benzidine; Pierce). After 10 min, the reactions were neutralized with 1.5 M H_2_SO_4_ (100 µL/well) and the level of binding was determined spectrophotometrically at 450 nm.

The final antibody titre of the llama to *N. kaouthia* venom was determined by doing an end-point titration ELISA. Serum from post-immune Day 134 (the last bleed collected) and pre-immune Day -7 (background) were serially diluted in blocking buffer (2% M-PBS) and incubated with venom-immobilized wells as previously described. The end-point antibody titre was determined as the dilution that gave 3× the absorbance (450 nm) value of the background (Pre-immune sera).

To determine if there was a HCAb immune response specific to α–Cbtx, pre-immune (Day -7) and post-immune (Day 110) llama sera were fractionated into the different IgG subclasses by differential absorption on protein G and protein A columns as described by Hamers-Casterman *et al*. [14] and van der Linden [35]. The resulting fractions, A1 (HCAb), A2 (HCAb), G1 (HCAb), and G2 (conventional IgG) were screened for specific binding to α–Cbtx by indirect ELISA as described for the polyclonal serum ELISA, with the following exception: Instead of using polyclonal serum, the fractions were serially diluted 1∶50 in blocking buffer with a starting concentration of 1.25 µg mL^−1^.

### V_H_H Library Construction and Selection of α–Cbtx V_H_H Binders

Total RNA was extracted from ca. 2×10^7^ leukocytes collected on Day 110 (Table 1) using a QIAamp RNA Blood Mini Kit (Qiagen). The V_H_H gene repertoire was amplified as previously described [36], [37], [38], [39], cloned into pMED1 phagemid vector [36], and transformed into *Escherichia coli* TG1 to create the library. The quality of the V_H_H library was estimated based on library size, insert ratio, and diversity of the V_H_H sequences. The V_H_H-displayed phage library was amplified and precipitated as described previously [40] with the exception of using helper phage M13K07 (New Englad Biolabs). For round 1 of panning, one well of a microtitre plate was coated with PBS (100 µL) and a second well was coated with 40 µg of α–Cbtx (100 µL). For rounds 2 and 3 of panning, the coating concentration of α–Cbtx was decreased to 20 and 5 µg, respectively. After o/n incubation at 4°C, both wells were blocked with 300 µL of 4% M-PBS at 37°C for 2 hr then washed 5×with PBS (300 µL). Amplified phage (100 µL) and 8% M-PBS (100 µL) were combined in a 0.5 mL tube and pre-incubated with rotation for 1 hr at RT. For subtractive panning of plastic binders, pre-incubated phage (100 uL) were first incubated in the PBS coated well for 1 hr at 37°C. After incubation, the content of the well was transferred to the α–Cbtx coated well and incubated for 2 hr at 37°C. Unbound phage were removed by washing 5, 8 and 12× with PBST (200 µL) for panning rounds 1, 2 and 3, respectively, and then washed 2× with PBS (200 µL). Bound phage were eluted with 200 µL of 100 mM triethylamine (TEA) and neutralized with 400 µL of 1 M tris-HCl (pH 7.4). Eluted phage were rescued, amplified and precipitated as described previously [40], and used in preparation for the next round of panning.

The progress of selection against immobilized α–Cbtx was monitored by performing an ELISA using polyclonal phage from each of three rounds of panning as well as with the unselected library phage. Wells from a Reacti-Bind™ maleic anhydride-activated polystyrene microtitre plate were coated with 100 µL of α–Cbtx (1 µg mL^−1^) at 4°C o/n. Wells were washed 3× with 200 µL of PBS (pH 7.4) to remove unbound antigen, and then blocked with 300 µL of SuperBlock (Pierce) at 4°C o/n. The remainder steps of the ELISA followed those as described previously [40].

A total of 46 colonies from the third round of panning were screened for α–Cbtx binding by monoclonal phage ELISA. Random colonies were grown and infected as described previously [40]. Microtitre wells were prepared as described for the polyclonal phage ELISA. The remainder steps of the ELISA followed those as described previously [40]. Phage with absorbance (450 nm) readings greater than 0.3 background were sequenced using the universal M13RP [39] primer at Laboratory Services, University of Guelph.

### Expression and Purification of α–Cbtx V_H_H Binders

For soluble expression of V_H_Hs, purified recombinant V_H_H-phagemid constructs were electroporated into *E. coli* strain HB2151, a non-suppressor strain. Single colonies were picked and transferred into 5 mL of 2× YT starter culture supplemented with ampicillin and 1% glucose. Cultures were grown o/n at 37°C while shaking at 220 rpm. For large-scale expression, 1 mL of starter culture was transferred into 1 L of 2× YT medium supplemented as described above and grown at 37°C while shaking at 220 rpm until the OD_600_ reached 0.6–0.7. The cell pellets were collected by centrifuging at 3,000×g and resuspended in fresh 2× YT medium (1 L) supplemented with ampicillin and 0.1% glucose. To induce soluble V_H_H expression, 1 mM isopropyl β-D-1-thiogalactopyranoside (IPTG) was added to the cultures. Cultures were grown at 26°C for 24 hr while shaking at 220 rpm and then the V_H_Hs were extracted as recently described in [41]. Soluble V_H_Hs were purified via their His_6_ tag using HisTrap HP metal affinity chromatography (GE Healthcare, Piscataway, NJ) by following the manufacturer's instructions. The eluted V_H_Hs were dialyzed (3,500 MW cutoff) at 4°C o/n against PBS (pH 7.4). The presence of the purified V_H_Hs was confirmed by standard SDS-PAGE and immunoblot.

### Engineering of the V_H_H2-Fc Antibody Construct

**Figure 1:**
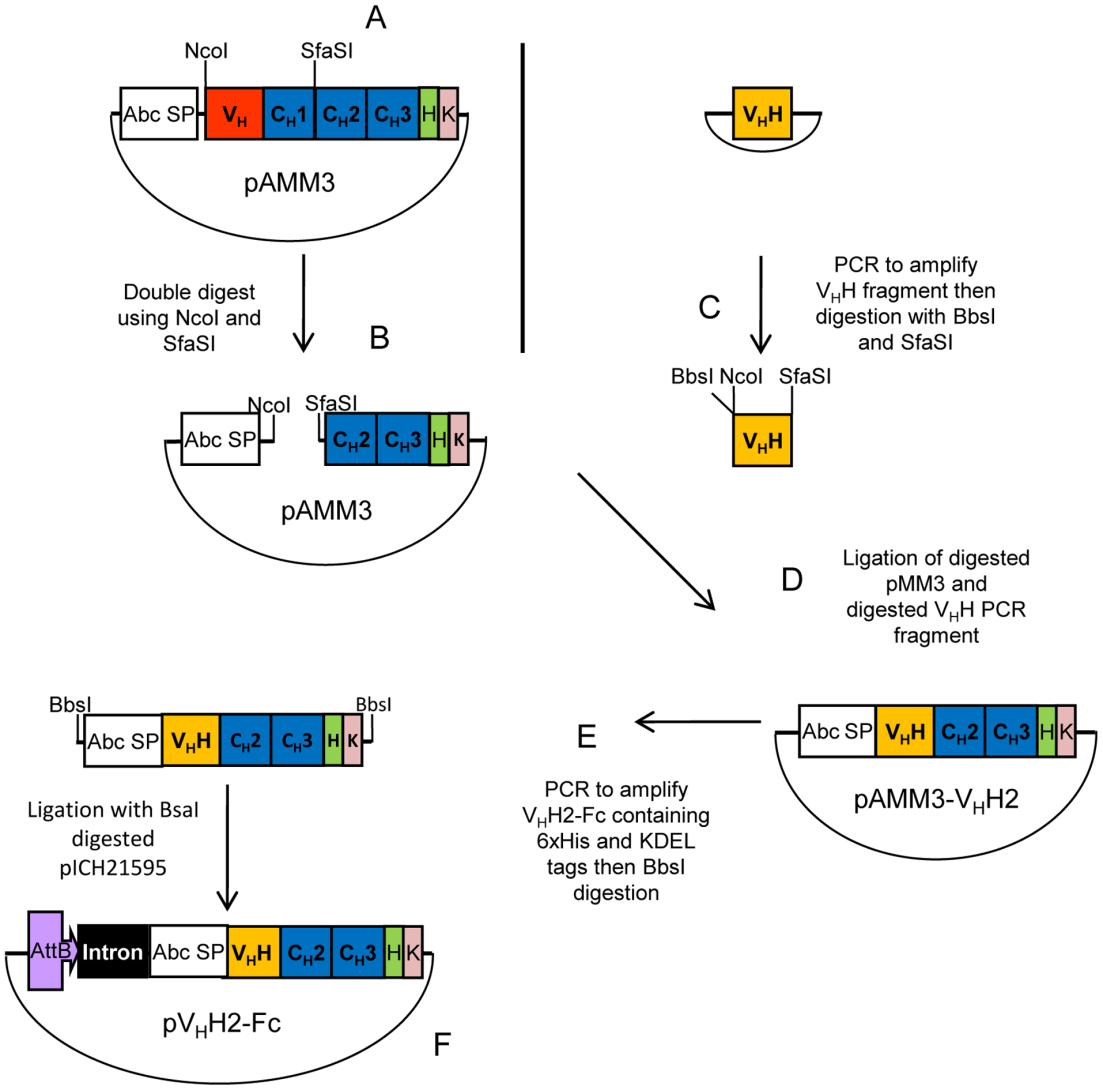
Schematic representation of the V_H_H2-Fc antibody cloning strategy. (A) pAMM3 was created by adding a *Sfa*SI restriction enzyme site to pMM3 using PCR mutagenesis. (**B**) pAMM3 was digested with *Nco*I and *Sfa*SI to remove V_H_ and C_H_1 domains and retaining the Fc of IgG1. (**C**) V_H_H C2 was PCR amplified and then digested with *Bbs*I and *Sfa*SI. (**D**) Digested pAMM3 and V_H_H C2 were ligated together thereby fusing the V_H_H with the Fc. (**E**) V_H_H2-Fc coding sequence was PCR amplified from pAMM3-V_H_H2, then digested with *Bbs*I. (**F**) Digested V_H_H2-Fc fragments were ligated with *Bsa*I-digested pICH21595 generating pV_H_H2-Fc construct. Restriction enzymes sites: *Nco*I, *Sfa*SI, *Bbs*I and *Bsa*I.

The magnICON® expression vectors, pICH20111, pICH14011, and pICH21595 were provided by Icon Genetics GmbH (Halle, Germany [42]). The cloning strategy for engineering the V_H_H2-Fc antibody is illustrated in Fig. 1. The V_H_H2-Fc antibody construct was assembled in pMM3 (a precursor of pMM29), a binary vector for the expression of the anti-*Pseudomonas aeruginosa* serotype O6ad human IgG1 HC [43]. A *Sfa*SI restriction enzyme (RE) site was inserted into pMM3 following codons encoding the first cysteine residue on the hinge region of the IgG1 HC, using the QuikChange® II Site-Directed Mutagenesis Kit (Stratagene, La Jolla, CA, USA) and primers pMM3 *Sfa*SI For and pMM3 *Sfa*SI Rev (Table 2), generating pAMM3 (Fig. 1A). pAMM3 was digested with *Nco*I and *Sfa*SI to remove the human variable heavy (V_H_) region and the constant heavy one (C_H_1) domain (Fig. 1B). The anti-α-Cbtx V_H_H C2 coding sequence was PCR-amplified using primers V_H_H *Bbs*I *Nco*I For and V_H_H *Sfa*SI Rev (Table 2) adding *Bbs*I and *Nco*I RE sites at the N-terminus and a *Sfa*SI RE site at the C-terminus (Fig. 1C). V_H_H C2 was digested with *Bbs*I and *Sfa*SI, resulting in *Nco*I and *Sfa*SI sticky ends (N.B., this was required due to an internal *Nco*I RE site). V_H_H C2 was inserted into pAMM3 in place of the V_H_ and C_H_1 regions, generating pAMM3-V_H_H2 (Fig. 1D). Primers V_H_H Fc *Bbs*I SS For and V_H_H Fc *Bbs*I HK Rev (Table 2), both containing *Bbs*I RE sites, were used to PCR-amplify the coding sequence from pAMM3-V_H_H2, which consisted of the *Arabidopsis* basic chitinase signal peptide (Abc SP), anti-α-Cbtx V_H_H C2, human IgG1 C_H_2 and C_H_3 regions, 6×His tag, and KDEL tag (Fig. 1E). The V_H_H2-Fc PCR product was digested with *Bbs*I, resulting in *Bsa*I-compatible sticky ends (N.B., *Bbs*I was used due to an internal *Bsa*I site within the V_H_H coding sequences) and inserted between the *Bsa*I sites in the MCS of the TMV-based 3′ vector pICH21595, generating 3′TMV-based V_H_H2-Fc (pV_H_H2-Fc) (Fig. 1F).

**Table 2:** Primers used for the construction of the V_H_H2-Fc antibody.

Primername	Nucleotidesequences
***PCR mutagenesis*** ***primers***	
pMM3 *Sfa*SI For	5′-CCAAAGTCATGCGCGATCGCCGACAAAACACATACTTG-3′
pMM3 *Sfa*SI Rev	5′-CAAGTATGTGTTTTGTCGGCGATCGCGCATGACTTTGG-3′
***V_H_H primers***	
V_H_H *Bbs*I *Nco*I For	5′-TTTTTTGAAGACGCCATGGCCCAGGTAAAGCTGGAGG-3′
V_H_H *Sfa*SI Rev	5′-TTTTTTGCGATCGCTGAGGAGACGGTGACCTGGGTCC-3′
***V_H_H Fc primers***	
V_H_H Fc *Bbs*I SS For	5′-TTTGAAGACAAAGGTATGGCTAAAACAAATCTCTTTTTA-3′
V_H_H Fc *Bbs*I HK Rev	5′-TTTGAAGACAAAAGCTCATTATAATTCATCCTTGTGATG-3′

### Plant Infiltration

To express the V_H_H2-Fc antibody, *Nicotiana benthamiana* plants were vacuum infiltrated with *Agrobacterium* strains containing pICH20111, pICH14011 and pV_H_H2-Fc according to the protocol described by Grohs et al. [44] with the following modification. A vacuum was applied for 2 min with pressure ranging from 0.5 to 0.9 bar and then slowly released [45]. The vacuum-infiltrated tissue was harvested, fresh weight recorded and stored at −80°C until extracted. Untreated *N. benthamiana* plants were used as a negative control.

### Extraction of Crude Plant Extracts

Frozen *N. benthamiana* tissue from 8 days post-infiltration (dpi) was ground under liquid nitrogen, then combined with three volumes of cold extraction buffer (40 mM phosphate buffer, 50 mM ascorbic acid and 10 mM ethylenediaminetetraacetic acid (EDTA) disodium salt). The combination was centrifuged at 12 000 rpm for 30 min at 4°C, the supernate collected and used for analyses. A Bradford Assay was used to determine the quantity of total soluble protein (TSP) present in the crude plant extract (cpe) with bovine serum albumin (BSA) as a standard. The cpe was analyzed using standard SDS-PAGE and immunoblotting techniques as described by Grohs et al. [44], with the following modifications. The immunoblot was probed with a 1∶5,000 dilution of protein A conjugated to HRP (Zymed Laboratories, San Francisco, CA, USA) and 1-Step™ TMB-Blotting substrate (Thermo Scientific, Fisher) was used to develop the immunoblot.

### Assessing *in planta* Expression Levels of the V_H_H2-Fc Antibody by Quantitative ELISA

For the quantification of the expression of V_H_H2-Fc antibody, crude plant extracts were analysed using a direct sandwich ELISA adapted from Vézina et al. [46] and Villani et al. [47]. A monoclonal anti-human IgG specific for the γ-chain (Sigma-Aldrich), was coated onto a Costar EIA/RIA 96-well plate (Corning Incorporated, Corning, NY, USA) at a concentration of 0.3125 µg mL^−1^ in PBS and incubated at 4°C o/n. The plate was blocked with 4% M-PBS at 4°C o/n then washed 5× with PBS-T. Two-fold serial dilutions of plant extracts, normalized for TSP, were added to the wells and incubated for 1 hr at 37°C. Two-fold serial dilutions (beginning with 100 ng) of a human IgG1 (Athens Research & Technology, Athens, GA, USA), normalized for TSP, were added to the plate as a standard. After washing, anti-human IgG (H+L)-HRP (Abcam, Cambridge, MA, USA) was added at a concentration of 1 µg mL^−1^ in M-PBS and incubated at 37°C for 1 hr. After washing 5× with PBS-T, the plate was developed, stopped and read as previously described. Three replicates from two different experiments were pooled to provide six replicates for each treatment. The data obtained were transformed to achieve linearity followed by an ANOVA to determine significant difference between treatments. Tukey’s Multiple Comparison test was used to determine significant differences among means.

### Purification of V_H_H2-Fc Antibody from Plants

Frozen biomass was ground under liquid nitrogen using a mortar and pestle, added to two volumes of cold extraction buffer and mixed to form a homogeneous slurry. The slurry was centrifuged at 12 000 rpm for 30 min at 4°C and the supernatant collected. Protein G-based affinity chromatography was used to purify the V_H_H2-Fc antibody from the cpe. The supernatant was filtered through two layers of Miracloth (Calbiochem CN biosciences, inc., La Jolla, CA, USA), then through a GF/C glass microfiber filter (Whatman, GE Healthcare), followed by filtration through 0.45 µm mixed cellulose ester (Sigma) and 0.22 µm nitrocellulose membranes (Millipore). The filtered supernatant was applied to a 5-mL Hi Trap Protein G HP column (GE Healthcare) equilibrated with 20 mM phosphate buffer. The V_H_H2-Fc antibody was eluted using a two-step elution with 0.1 M citrate pH 3.5 followed by 0.1 M glycine-HCl pH 2.3 and in both cases the eluates were immediately neutralized with 1 M Tris-HCl pH 8.5. The eluates were dialyzed against 20 mM phosphate buffer o/n at 4°C in a 10 kDa MW cutoff dialysis tube, after which the retentate was filtered through a 0.22 µm nitrocellulose membrane and stored at 4°C. An additional Protein G-based affinity chromatography step was required to ensure that all impurities were removed. The antibodies were concentrated using Amicon Ultra-4 centrifugal filter devices (Millipore) with a 10 kDa MW cutoff. After concentration, a Bradford Assay was used to quantify the amount of antibody; BSA was used as a standard. The purity of the V_H_H2-Fc antibody was evaluated using standard SDS-PAGE and immunoblotting in which the membrane was probed with a polyclonal anti-human IgG (γ-specific) conjugated to alkaline phosphatise (AP). Densitometry was used to measure the intensity of the bands on the immunoblot using Bio-Rad Quantity One Software.

For N-terminal sequencing, the plant-purified V_H_H2-Fc antibody preparation was electrophoresed on a 12% SDS-PAGE under reducing conditions then transferred to a Sequi-Blot PVDF membrane (Bio-Rad). The membrane was stained with Coomassie R-250 stain. N-terminal sequencing (Edman degradation) was performed at the Hospital for Sick Children Research Institute in the Advanced Protein Technology Centre (University of Toronto).

### Surface Plasmon Resonance (SPR) Analyses

Binding kinetic experiments were performed by SPR using a Biacore 3000 instrument (GE Healthcare). For monovalent kinetic analysis, approximately 137 resonance units (RUs) of α–Cbtx were immobilized on a CM5 research grade sensor chip (BIACORE) in 10 mM acetate buffer pH 4.5 using an amine coupling kit (GE Healthcare) supplied by the manufacturer. Before SPR analysis, purified V_H_Hs were subjected to size exclusion chromatography using a Superdex™ 75 column (GE Healthcare). V_H_H monomers were passed over the coated sensor chip in HBS-EP running buffer [10 mM HEPES, pH 7.4, 150 mM NaCl, 3 mM EDTA, 0.005% surfactant P-20 (GE Healthcare)]. The four V_H_Hs (C2, C19, C20 and C43) were injected at concentrations ranging from 2.5 to 30 nM, 6.25 to 150 nM, 1 to 32 nM and 5 to 160 nM, respectively. All experiments were conducted at RT at a flow rate of 40 µL min^−1^. The surface was regenerated with HBS-EP buffer or with 50 mM NaOH (2 µL) when C2 was injected. Data were analyzed with BIAevaluation 4.1 software (GE Healthcare).

For kinetic analysis of bivalent antibodies, approximately 4590 RUs of a control IgG and 3918 RUs of V_H_H2-Fc antibody were immobilized in 10 mM acetate buffer pH 3.5 to a sensor chip as previously described. α–Cbtx (10 nM) was passed over the antibody-coated sensor chip in HBS-EP buffer for 2 min and allowed to dissociate for 15 min at a flow rate of 40 µL min^−1^. The dissociation rate constant (*k*_d_) were determined using *k*_d_ fitting and kinetic analysis assuming a 1∶1 binding model.

### *In vivo* Neutralization of α–Cbtx-induced Lethality

Since there are already published data on the *in vivo* toxicity of purified α–Cbtx in mice (LD_100_ = 100 µg/kg, i.v.) [48], a small pilot study was carried out to ensure the toxicity of our α–Cbtx stock. Mice (n = 2/group; 22–30 g) were intraperitoneally (i.p.) administered with different doses of α–Cbtx (administration volume of 150 µL in sterile PBS, pH 7.4). Mice were closely monitored for 24 hr for signs of early neurotoxicity (e.g. change in activity level, impaired movement), abnormal respiration (e.g. slow, shallow*, laboured*), severe neurotoxicity (e.g. paralysis*), change in body temperature (≤34°C*) and other common ‘sick mouse’ conditions (e.g. wasp-waisted, ruffled coat, dehydrated, sunken eyes). Conditions marked with an asterisk (*) were considered the end-point of this study for the animal and it was humanely euthanized. Mice were counted as live/dead after 24 hr. To confirm the lethal dosage, a larger group of mice (n = 6, repeated twice for a total of 12 mice) was i.p. administered the lowest dose that caused 100% fatality. This pilot study showed that 4 µg of our α–Cbtx stock caused 100% mice fatality within 24 hr and this dose was considered as the LD_100_ for our experiments (data not shown).

To evaluate the ability of our V_H_Hs and the V_H_H2-Fc antibody to protect mice from α–Cbtx challenge, mice (n = 6 per group) were i.p. administered the LD_100_ concentration of α–Cbtx pre-incubated for 1 hr at 37°C with various molar ratio (MR) of the selected V_H_Hs (C2, C19, C20, C43) or with the V_H_H2-Fc antibody. For example, 1×MR indicates 1∶1 Ab:toxin MR [e.g. 8.5 µg of V_H_H (Mr 16.6 kDa): 4 µg α–Cbtx (Mr 7.8 kDa)]. When possible, the lowest amount of the antibody that offered 100% survival was administered. Control treatments included commercial equine *N. kaouthia* antivenom (10×w/w) (Queen Saovabha Memorial Institute, Bangkok, Thailand) pre-incubated with α–Cbtx, α–Cbtx (no antibody), V_H_H C2 (no toxin) and a non-specific V_H_H P2 (negative control; anti-*Pythium aphanidermatum* V_H_H P2) pre-incubated with α–Cbtx. Mice were monitored as previously described for 24 hr. The number of live/dead mice were counted and expressed as percent survival.

As V_H_H C2 was the V_H_H that offered the best *in vivo* protection, it was chosen for further characterization. For the mock envenomation experiment, mice were administered α–Cbtx i.p. followed by C2, 15 or 30 min post-intoxication. Mice were monitored as previously described. The number of live/dead mice were counted and expressed as percent survival. The neutralizing capacity of C2 was evaluated by administering increased amounts of α–Cbtx (1x, 2x and 4x LD_100_) pre-incubated with 0.75x, 1x, or 2x MR of V_H_H C2. Mice were monitored as previously described. The number of live/dead mice were counted and expressed as percent survival.

## Results

### Humoral Response

**Figure 2:**
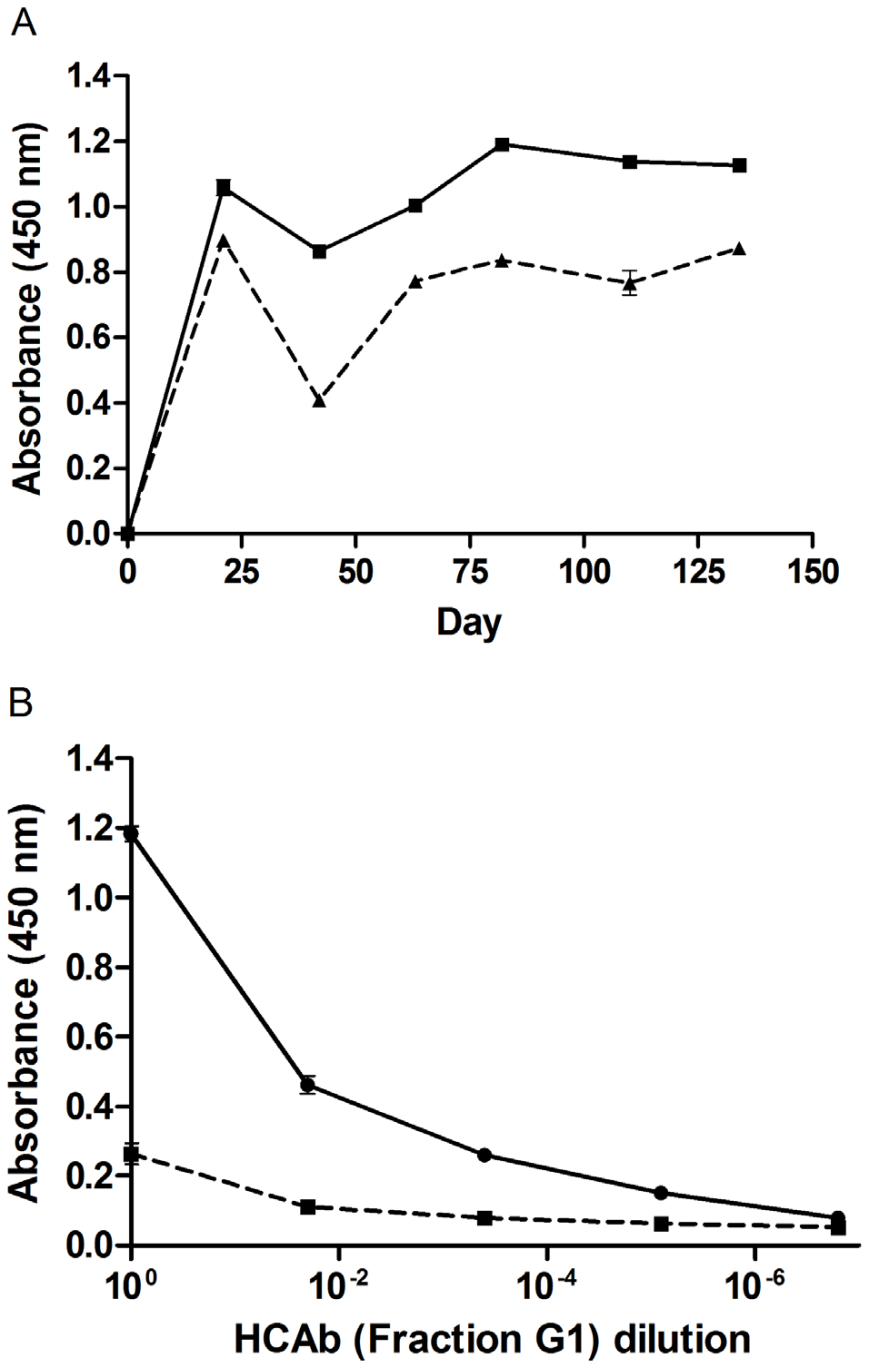
Llama’s polyclonal and HCAb immune response. (A) Time-course ELISA. The llama’s humoral response to immunization with *N. kaouthia* venom was monitored by indirect ELISA. The llama showed a rapid and strong response to *N. kaouthia* venom (solid line) and α–Cbtx (dash line) after receiving two injections (i.e., by Day 21; see immunization schedule Table 1); however, the response decreased abruptly after the third injection (Day 42). For subsequent immunizations, the adjuvant (TiterMax™ Classic adjuvant) was replaced with IFA and the humoral response increased. (**B**) HCAb response to α–Cbtx. Pre-immune (Day -7; dash line) and post-immune (Day 110; solid line) llama sera were fractionated into conventional IgG and HCAb as described by [14] and [35]. HCAb (Fraction G1; 1.25 µg mL^−1^) was serially diluted (1∶50 v/v) and its specific binding to α–Cbtx was detected by indirect ELISA. Numbers are the average of triplicates. SEMs are shown with bars; when bars are not shown they are smaller than the symbol.

The humoral response to *N. kaouthia* venom and α–Cbtx were monitored over the course of the llama immunization schedule (Fig. 2A). The llama elicited a rapid temporal response to venom and α–Cbtx by post-immune Day 21, after receiving two injections of crude *N. kaouthia* venom (See immunization schedule Table 1). Thereafter, we observed a sharp decrease in the humoral response to both venom and α–Cbtx at Day 42. When we replaced TiterMax™ Classic adjuvant with IFA in subsequent injections, we observed that the humoral response to the venom and α–Cbtx also increased. The final antiserum titre to *N. kaouthia* venom was assessed by titrating the serum from the last bleed (Day 134) against immobilized *N. kaouthia* venom in an indirect ELISA. The serum dilution that corresponded to 2x the value of the background (Pre-immune sera) was determined as 3.0×10^5^ (Fig. S1). Before proceeding with construction of V_H_H phage-display library, we confirmed that there was a specific HCAb response to α–Cbtx (Fig. 2B).

### Construction of V_H_H Library and Selection of α–Cbtx Binders

The size of the library was estimated to be 5.0×10^9^ individual transformants with 84% containing a V_H_H coding sequence (data not shown). The library is therefore degenerate since it was constructed from total RNA extracted from ca. 2×10^7^ leukocytes. Sequence analysis showed good diversity among the twenty random clones sequenced with few conserved amino acid residues in the CDR regions (data not shown). In particular, the CDR3 region varied considerably in terms of the amino acid composition and length. Furthermore, this library contained a good repertoire of the different V_H_H subfamilies, except for V_H_H subfamily 4, which is naturally more rare [49].

**Figure 3:**
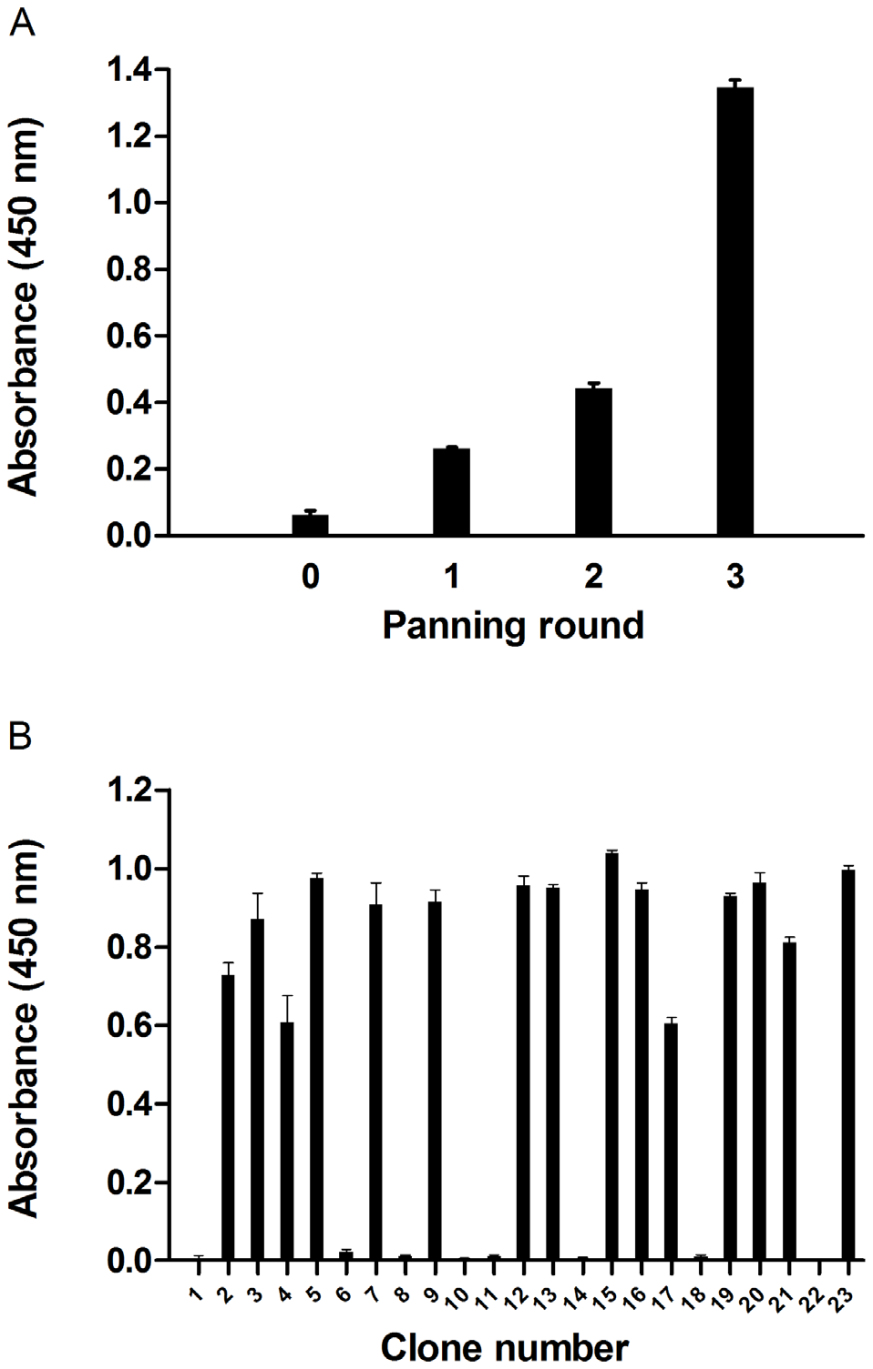
Selection of α–Cbtx V_H_H-displayed phage binders. (A) Polyclonal phage ELISA. The progress of selecting phage-V_H_Hs specific to α–Cbtx over the three rounds of panning was assessed by doing a polyclonal phage ELISA. Eluted phage (amplified; 100 µL of 10^11 ^pfu mL^−1^) from each round of panning were incubated with immobilized α–Cbtx (1 µg mL^−1^) and detected with anti-M13 antibody conjugated to horseradish peroxidise. (**B**) Monoclonal phage ELISA. Forty-six (not all data shown) monoclonal phage from the 3^rd^ round of panning were randomly selected and their specificities to α–Cbtx were assayed by indirect ELISA with anti-M13 antibody conjugated to horseradish peroxidase. Absorbance values at 450 nm are the mean of triplicates with background (blocked wells with no α–Cbtx) subtracted. SEMs are shown with bars.

The progress of selection against immobilized α–Cbtx was monitored by performing an ELISA using polyclonal phage from each round of panning and with the unselected library phage as a control (Fig. 3A). After three rounds of panning, the ELISA signal was at least 3 and 10 times greater than that from round 2 and the unselected library, respectively, indicating enrichment for clones specific to α–Cbtx. To identify specific α–Cbtx V_H_H binders, 46 individual clones from the 3^rd^ round of panning were screened by monoclonal phage ELISA. About 60% of the monoclonal phage (27/46) showed specific binding to α–Cbtx (Fig. 3B, not all data shown), and the positive clones were sent for DNA sequencing.

### Sequence Analysis of Round 3 Anti-α–Cbtx V_H_H Clones

**Figure 4:**
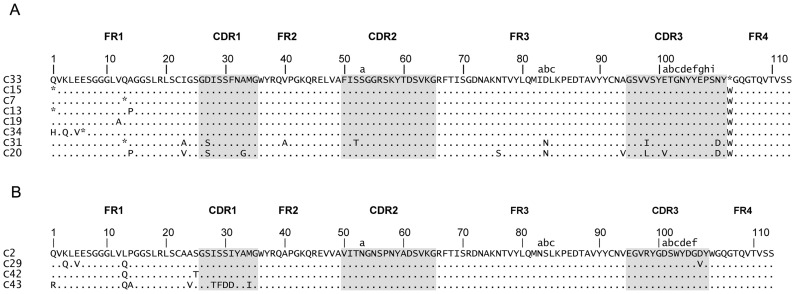
Amino acid sequence alignment of anti-α–Cbtx V_H_H binders. The clones were categorized in either (**A**) Cluster I or (**B**) Cluster II based on their sequence homologies. Framework regions (FR) and complementarity determining regions (CDR) (shaded in grey) as well as amino acid insertions (52a, 82a, b, c, 100a, b, c, d, e, f, g, h, i) are defined according to the Kabat numbering system [52]. The dots in the sequences indicate amino acid identity that is the same as in C33 (Cluster I) or C2 (Cluster II). All clones belong to V_H_H Subfamily 2 [49]. An asterisk (*) represents an amber stop codon (TAG) mutation. However, TG1 *E. coli*, which was used during panning, has the *supE* amber suppressor mutation (now known as *glnV* gene for tRNA^gln^ gene) that inserts a glutamine (Q) when stop codon readthrough occurs [53]. Therefore, a glutamine (Q) would have been placed were asterisks are marked, thus allowing these clones to survive the panning process.

Sequence analysis revealed that the 3^rd^ round of panning generated several unique α–Cbtx binders. As revealed by a multiple sequence alignment (MSA) of the predicted amino acid sequences, many clones shared high identity with at least one amino acid substitution (Fig. 4). Moreover, based on CDR homology and CDR3 length, two distinct groups of α–Cbtx binders are apparent, hereafter named Cluster I and Cluster II.

The eight clones (C33, C46, C7, C13, C19, C34, C31, C20) that form Cluster I are characterized by a CDR3 length of 17 amino acid residues with the following consensus motif: GSV(V/L/I)SY(E/V)TGNYYEPS(N/D)Y. All these binders have identical CDR2 regions (FISSGGRSKYTDSVK), and a well conserved CDR1 (consensus motif: G(D/S)ISSFN(A/G)MG). In contrast to Cluster I, clones that form Cluster II (C2, C29, C43, and C42) have a shorter CDR3 region with 14 amino acid residues, which has the following highly conserved consensus motif: EGVRYGDSWYDG(D/V)Y. Like Cluster I, clones among Cluster II share an identical CDR2 with the sequence VITNGNSPNYADSVKG. Furthermore, all the clones from this group have a conserved CDR1 with the sequence GSISSIYAMG, except C43 which has a unique CDR1. All the α–Cbtx binders isolated (Cluster I and Cluster II clones) from this immunized library belonged to V_H_H subfamily 2 (V_H_H-2) [49].

### α–Cbtx Binders Selected for Further Characterization

Four α–Cbtx V_H_H clones that showed good binding by ELISA and did not contain amber stop codon mutations were selected for further characterization; two from Cluster I (C19 and C20) and two from Cluster II (C2 and C43). As shown in Fig. 4, C2 and C43 differ by nine amino acid residues, which are located in FR1 and CDR1. C19 and C20 differ by 10 amino acid residues, which are located in different regions. Immunoblot analysis (Fig. S2) showed that the four selected clones were successfully expressed in 1-L *E. coli* HB2151 cultures, and purified from the periplasmic fractions using IMAC. Based on the predicted amino acid composition along with the His_6_ detection/purification tag, the theoretical MWs of the V_H_Hs were predicted to be ca. 16.6 kDa.

### Accumulation of V_H_H2-Fc Antibody *in planta*

Whole-plant vacuum infiltrations were used to obtain large quantities of the V_H_H2-Fc antibody. Three plants were infiltrated and subsequently analyzed to determine expression. Equal amounts of TSP from the three plants were pooled to create a sample representing V_H_H2-Fc antibody. The pooled sample was analyzed by 12% standard SDS-PAGE and immunoblot (Fig. S3). The banding pattern of the plant-produced V_H_H2-Fc antibody was similar to the pattern of the llama HCAb control that was spiked into cpe from wild type plants. Specifically, the plant-produced assembled V_H_H2-Fc antibody and unassembled V_H_H2-Fc antibody migrated similarly to the llama HCAb control (Fig. S3B). Additional bands were observed just above the 25 kDa molecular marker, suggesting the V_H_H2-Fc antibody may have degraded during the extraction process (Fig. S3B, see asterisks). A quantitative ELISA determined that the plants expressed an average of 129.50 (SEM 44.8) mg of antibody per kilogram of fresh leaf tissue (mg kg^−1^ FW) (data not shown).

### Purification of V_H_H2-Fc Antibody

**Figure 5:**
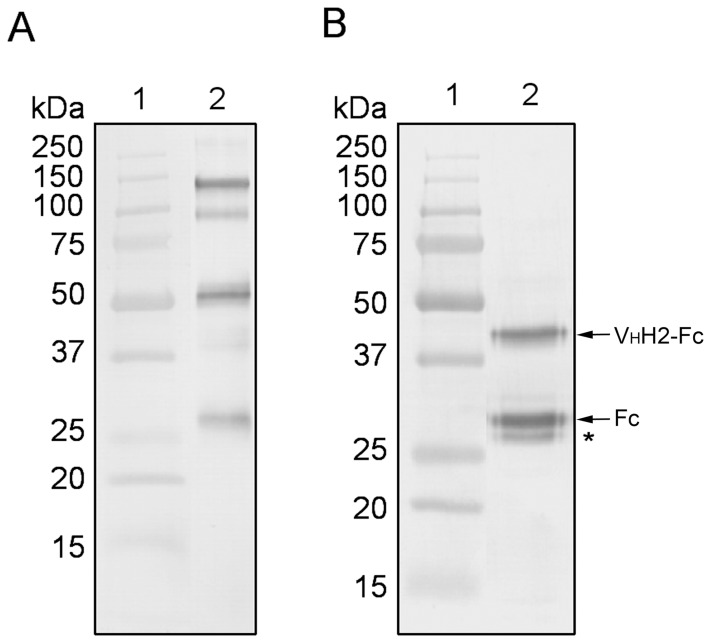
Immunoblots of purified V_H_H2-Fc antibody from *N. benthamiana*. V_H_H2-Fc antibody was purified twice using Protein G-based chromatography, separated using standard 12% SDS-PAGE under non-reducing (**A**) and reducing (**B**) conditions, electrotransferred to PVDF membranes and probed with a polyclonal anti-human IgG (γ-specific) conjugated to AP. Lane 1, molecular weight standard; Lane 2, 100 ng of V_H_H2-Fc antibody. See text for an explanation of the asterisk.

Protein G-based chromatography was used to purify the V_H_H2-Fc antibody from filtered plant extract. A second Protein G step was performed to increase purity of the V_H_H2-Fc antibody. Standard SDS-PAGE was used to resolve 100 ng of the purified V_H_H2-Fc antibody under non-reducing and reducing conditions, and then transferred to a membrane for immunoblot analysis (Fig. 5). Under non-reducing conditions V_H_H2-Fc antibody did not strictly resolve according to the predicted MW (80.6 kDa), which is typical for large multimeric molecules (Fig. 5A; lane 2). The band at ∼150 kDa is considered to be the assembled V_H_H2-Fc antibody, and the band above 50 kDa is considered to be assembled Fc region that was cleaved from the V_H_H2-Fc antibody. The additional bands at ∼100 kDa and above 25 kDa are considered to be degradation products. However, under reducing conditions the V_H_H2-Fc antibody resolved more accurately according to its predicted MW (Fig. 5B; lane 2). According to N-terminal sequencing, the band just below 50 kDa corresponds to reduced V_H_H2-Fc antibody (∼40 kDa); the prominent band below 37 kDa was identified to contain only the Fc region; the weak band is considered to be a degradation product (see asterisk). Based on densitometric analysis of lane 2 of Figure 5B, the intact V_H_H2-Fc represents 39% of the sample (Table 3).

**Table 3:** Densitometric analysis of the plant-purified V_H_H2-Fc antibody product (the three bands of Fig. 5B, lane 2) evaluated using the densitometry feature of Quantity One Software.

	Trace Quantity^b^	Relative Quantity
**V_H_H2-Fc**	845.354	0.39
**Fc only**	972.254	0.44
**Degradation Product**	368.301	0.17
**Total Trace Quantity**	2185.909	1.00

b Trace Quantity is the quantity of a band as measured by the area under its intensity profile curve, and Relative Quantity is the quantity of a particular band in a lane expressed as percentage of the total quantity of all the bands in the lane.

### Kinetic Analysis

**Figure 6:**
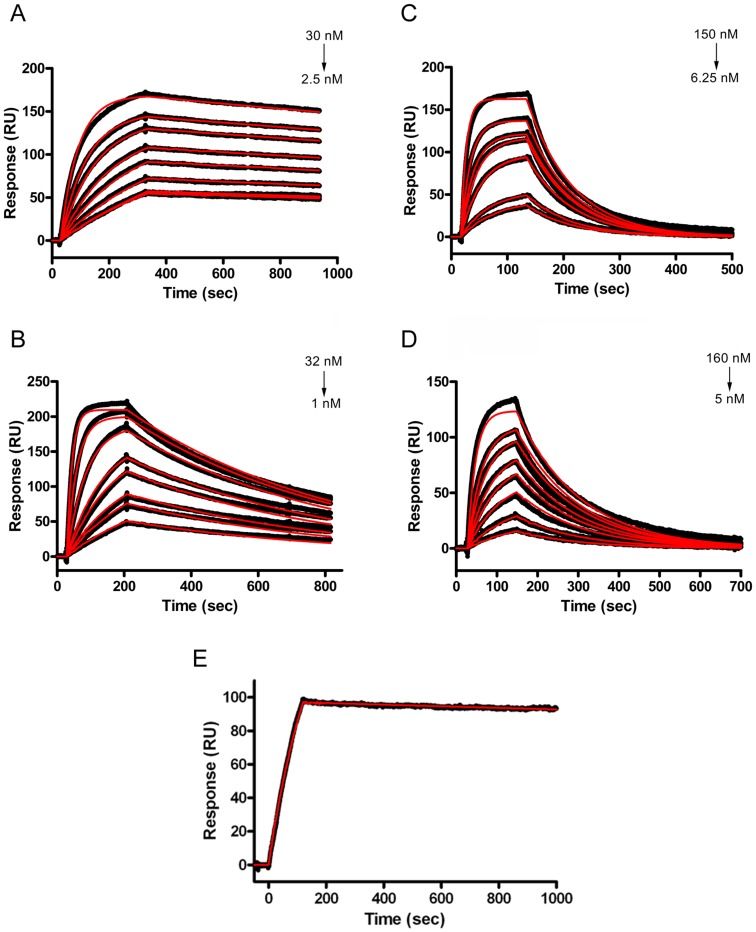
The V_H_Hs and the V_H_H2-Fc antibody bind to α–Cbtx with high affinity. Overlaid SPR sensograms of anti-α–Cbtx V_H_Hs C2 (**A**), C20 (**B**), C19 (**C**), and C43 (**D**) binding to immobilized α–Cbtx (**A–D**) and α–Cbtx binding to immobilized V_H_H2-Fc antibody (**E**) are shown. In experiments involving monomeric V_H_Hs, α–Cbtx was immobilized on a CM5 chip, and V_H_Hs were passed over the surface at concentration indicated. In experiments involving bivalent V_H_H2-Fc antibody, the V_H_H2-Fc antibody was immobilized on a CM5 chip and 10 nM α–Cbtx was passed over the surface. The data sets were analyzed by global fitting with a one-to-one interaction model (BIAevaluation software). Rate and affinity constants are summarized in Table 4.

The affinities of the four selected V_H_H clones for α–Cbtx were measured by surface plasmon resonance (SPR) (Fig. 6). The rate and affinity constants were determined by global fitting of the data set with a one-to-one interaction model. Equilibrium dissociation constants and kinetic rate constants are summarized in Table 4. All four V_H_H clones exhibited high affinity (i.e. low nM) to immobilized α–Cbtx. In particular, α–Cbtx had the highest affinity for C2 with a sub-nanomolar K_D_ value of 0.4 nM. The lower K_D_ of C2 is mostly due to its slow dissociation rate constant of 10^−4^ s^−1^, compared to 10^−2^–10^−3^ s^−1^ for the other clones. C20 had the fastest association rate constant; however, its dissociation was faster (i.e. greater) than that of C2. C19 and C43 had 60-fold and 25-fold lower affinity than that of C2, respectively. The V_H_H2-Fc antibody showed very good binding to α–Cbtx, with a dissociation rate constant (*k*_d_) of 5×10^−5^ s^−1^, as determined using a 1∶1 binding model (Fig. 6E).

**Table 4:** Kinetic and affinity constants of the monovalent V_H_Hs and the bivalent V_H_H2-Fc antibody against α–Cbtx as measured by surface plasmon resonance (SPR).

Antibody	K_D_ (nM)^c^	*k*_on_ (M^−1^s^−1^)	*k*_off_ (s^−1^)
**V_H_H C2**	0.4	5.2×10^5^	1.9×10^−4^
**V_H_H C43**	24	2.9×10^5^	6.8×10^−3^
**V_H_H C19**	25	5.8×10^5^	1.0×10^−2^
**V_H_H C20**	1	1.8×10^6^	1.7×10^−3^
**V_H_H2-Fc antibody**	N/A	N/A	5.0×10^−5^

c Equilibrium dissociation constants K_D_ (*k*_off_/*k*_on_), association (*k*_on_) and dissociation (*k*_off_) rate constants.

### *In vivo* Mouse Challenge

**Figure 7:**
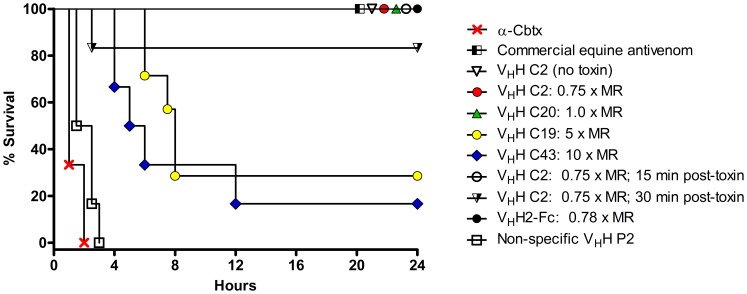
*In vivo* neutralization of α–Cbtx-induced lethality in mice with different α–Cbtx-specific V_H_Hs and a V_H_H2-Fc antibody. The lowest antibody:toxin molar ratio (MR) required to protect 100% of mice, or the highest antibody:toxin dose tested if 100% protection could not be obtained, are shown. Mice receiving commercial equine *N. kaouthia* antivenom (10×w/w) served as a positive control while non-specific V_H_H P2 pre-incubated with α–Cbtx served as negative control. With the exception of the post-toxin administration experiments, the toxin and the antibody preparations were pre-incubated for 1 hr at 37°C before i.p. administration to mice.

The ability of our V_H_Hs (C2, C19, C20 and C43), and V_H_H2-Fc antibody, to offer protection against α–Cbtx challenge was evaluated in an *in vivo* mouse challenge. In the absence of specific antibodies, mice administered i.p. with a lethal dose of α–Cbtx (4 µg) rapidly showed signs of neurotoxicity, and 100% mice died within the first 2–5 hours post-toxin injection (Fig. 7). Commercial cobra antivenom (positive control) showed full protection, while non-specific V_H_H P2 (negative control; anti- *Pythium aphanidermatum* V_H_H) did not offer any protection. When co-administered with α–Cbtx, all the specific V_H_Hs allowed prolonged or complete survival against α–Cbtx challenge (Fig. 7). The most efficacious V_H_Hs were C2 and C20 which neutralized α–Cbtx at 0.75× and 1.0× MR (molar ratio) of V_H_H:toxin, respectively. While C19 and C43 offered some neutralizing activity, 100% protection could not be attained with the amount of purified protein available at the time. The V_H_H2-Fc antibody, at a 0.78× MR, showed complete neutralization of α–Cbtx with 100% survival (Fig. 7).Since C2 was the most efficacious V_H_H, it was chosen for further testing. For a mock envenomation test, C2 administered 15 or 30 minutes post-toxin injection provided 100% (6/6) and 83% (5/6) survival, respectively (Fig. 7). In addition, the neutralizing capacity of C2 was evaluated by challenging mice with increased dosage of α–Cbtx and treating with various C2:toxin molar ratio (Table 5). The data show that the neutralizing capacity of C2 at 1, 2 and 4× the LD_100_ α–Cbtx is 0.75, 1 and 2× on a molar basis, respectively.

**Table 5:** *In vivo* neutralizing capacity of V_H_H C2 against increasing lethal doses (LD_100_) of α–Cbtx.

	C2:toxin molar ratio (MR)^d^		
LD_100_	0.75×	1×	2×
**1×**	6/6	6/6	-
**2×**	4/6	6/6	-
**4×**	2/6	4/6	6/6

d V_H_H C2 was pre-incubated with the indicated molar ratio of V_H_H C2 for 1 hr at 37°C before i.p. administration to mice.

## Discussion

Since its first use in the late 1800s by Calmette, passive immunotherapy using conventional antivenoms remains the only specific treatment for snake bite envenomation [4]. Although conventional antivenoms help increase survival rate, there are many problems associated with their use including *i)* their poor neutralization of toxins in deep tissues, *ii)* the adverse reactions they elicit (anaphylaxis, serum sickness), and *iii)* their limited supply [4]. In fact, Gutiérrez *et al*. [3] have categorized snake bite envenomation as a major neglected disease of the 21^st^ century. There is a need for more efficacious, safer and widely distributed therapeutics for snake envenomation.

In recent years, members of the camelidae (camels, llamas, alpacas) have been suggested for antivenom production. Similar to horses, camelids are docile and are able to yield large volumes of serum, and camel species are better suited than horses to live in hot, tropical and desert areas where snakes are most prevalent. In addition, camelid antibodies may represent a safer alternative to conventional equine and ovine antivenom antibodies because they are less immunogenic and therefore less likely to elicit adverse effects in patients [7], [29]. Further, camelid-derived single-domain antibody fragments (V_H_Hs) possess several attractive physico-chemical properties that may make them better therapeutic reagents for the treatment of envenomation. Due to their low molecular mass (∼15 kDa), V_H_Hs penetrate tissue compartments more readily than conventional antibody fragments [25], [27], [50] and, therefore, may protect victims from the tissue-damaging effects of venom toxins. In addition, V_H_Hs often have a longer CDR-3 loop that protrudes from the paratope allowing it to target cryptic enzyme clefts that are often nonreactive to conventional V_H_s [15], [16]. Finally, V_H_Hs have enhanced stability and solubility and are easily expressed and purified from *E. coli* or yeast expression systems, which may make production cheaper and more efficient.

We previously isolated three V_H_Hs from a naïve llama phage-display V_H_H library against α–Cbtx, the most potent α–neurotoxin from the venom of *N. kaouthia* [12]. Since their affinities were too low for therapeutic use (2–3 µM), we set out, in this study, to isolate higher affinity single-domains from a hyperimmune library. We immunized a llama with crude *N. kaouthia* venom instead of purified α–Cbtx (see immunization schedule Table 1). Our immunization scheme was inspired by a “low dose, low volume, multi-site immunization” protocol described by Chotwiwatthanakun *et al*. (2001) that showed potent horse IgG titres for antivenom production. However, we modified this protocol in terms of adjuvant used, time interval between injections, total number of injection sites, and injection volumes. Our llama, which was immunized with up to 2 mg of crude *N. kaouthia*, did not suffer from any visible signs cobra envenomation or local reaction at the injection sites. After receiving the first boost (post-immune Day 21; total of 2 injections), the llama generated a rapid humoral response to *N. kaouthia* venom and more specifically to α–Cbtx (Fig. 2A). Others have also observed a rapid rise in IgG titres following venom-immunzation in camelids and horses [8], [17], [33], [34]. Thereafter, we observed an abrupt decrease in the humoral response after the second boost (Day 42, total of 3 injections). In subsequent injections, we decided to replace the adjuvant (TiterMax™) with incomplete Freund's adjuvant (IFA), and consequently observed the humoral response to increase again (Fig. 2). One report has shown that α–Cbtx elicits its highest titres rapidly in the immunization schedule and that after reaching a plateau, titres slowly decrease even after continued injections [34]. Our llama’s final (last bleed collected) antiserum titre to *N. kaouthia* was 3.0×10^5^ (Fig. S1) and is very comparable to that observed by Cook et al. [17] in camels immunized with *N. nigricollis* venom.

After detecting a llama HCAb immune response against α–Cbtx (Fig. 2B), we constructed a large phage-displayed V_H_H library of 5.0×10^9^ clones with 84% containing a V_H_H coding sequence. Sequence analysis revealed that the 3^rd^ round of panning generated several unique V_H_H sequences. Based on CDR homology and CDR3 length, the V_H_H clones were grouped into either Cluster I or Cluster II (Fig. 4). Binders clustered together share high CDR sequence identity; however, little CDR homology is shared between these two clusters. Furthermore, the α–Cbtx binders from Cluster I have a CDR3 length of 17 amino acid residues while those from Cluster II have 14 residues.

Several differences were also noted when these V_H_Hs were aligned (alignment not shown) against those we previously isolated from a naïve library [12]. The naïve binders all belong to V_H_H subfamily 1 while the immune ones belong to V_H_H subfamily 2 [49]. As expected, the immune V_H_Hs share little CDR homology with the naïve ones, since they had the opportunity to go through natural affinity maturation. For instance, immune V_H_Hs have a shorter (14 or 17 residues) CDR3 region than the naïve ones (18 or 19 residues). This result is consistent with the literature since V_H_H subfamily 2 have on average a shorter CDR3 region than other subfamilies [49]. We chose two clones from Cluster I (C19 and C20) and two clones from Cluster II (C2 and C43) for further characterization. The clones were expressed in 1 L *E. coli* cultures (yields: 12–18 mg L^−1^) and purified from the periplasmic fractions using IMAC.

Kinetic analysis by SPR revealed that high affinity α–Cbtx binders were isolated from the immune library (Table 4, Fig. 6). In particular, C2 had the strongest affinity for α–Cbtx with K_D_ value of 0.4 nM. Despite having identical CDR2 and CDR3, C43 had 60-fold less affinity (K_D_ = 24 nM) than C2 to α–Cbtx (Table 4). It is in the CDR1 that they differ the most (5/10; 50% identity) with some FR1 substitutions as well. The differences in binding affinities to α–Cbtx among the clones suggest that the CDR1 is instrumental in binding α–Cbtx, at least for these two V_H_Hs. Accordingly, Vu *et al*. [29] have previously suggested that, in contrast to human and mice V_H_s, amino acid residues from the CDR1 of camelid V_H_Hs play a critical role in antigen-binding. C19 and C20 also had strong affinities to α–Cbtx with K_D_ values of 25 nM and 1 nM, respectively. Furthermore, our immune α–Cbtx V_H_H binders had ∼1000-fold higher affinity compared to those we previously isolated from a naïve V_H_H library (2–3 µM) [12]. Although immune libraries are cumbersome and time-consuming to construct, our result show that it was necessary to construct an immune library to isolate high affinity binders against α–Cbtx that offer full *in vivo* protection.

When tested in an *in vivo* mouse challenge, all of our isolated V_H_Hs showed neutralizing activity against lethal doses of α–Cbtx (Fig. 7). There was an inverse correlation between the neutralizing ability of each V_H_H and its K_D_, i.e., the highest affinity binder C2 (K_D_ = 0.4 nM) was able to neutralize the toxin at the lowest dose of 0.75× MR antibody:toxin. In terms of weight/weight (w/w), 6.38 µg of V_H_H C2 completely neutralized 4 µg of α–Cbtx, thus representing 1.6× w/w. In comparison, a previous report showed that their most efficacious human naïve scFv (HuScfv clone #24) against α–Cbtx was only able to protect 33% of mice when administered at 10× w/w (2.65 µg HuScFv/0.265 µg α–Cbtx), and full protection was not attained even when administered at a much higher dose (83.9 µg HuScFv/0.265 µg α–Cbtx) with 83% survival for 6 hours [21]. However, we did observe a discrepancy between our lethal dose (LD_100_ of 4 µg, this study) and theirs (LD_100_ of 0.265 µg, [21]). Our lethal dose is in accordance with the toxicity reported by Karlsson and Eaker [48] and with the product information sheet supplied with our purchased α–Cbtx (LD_100_ = 100 µg kg^−1^).

Despite their improved tissue permeability, the clinical use of V_H_Hs as the only reagent in antivenom preparations would likely be limited by their rapid clearance from the body. In fact, if V_H_Hs would likely be eliminated from the body before venom toxins reached circulation, signs of recurrence of evenomation after initial successful therapy may arise as has been observed with treatment with Fab antivenoms [4]. Since several recent reports have suggested that the ideal antivenom should contain a combination of low and high molecular weight antibody reagents [8], [12], [30], [32], we created a V_H_H-Fc antibody by fusing our highest affinity α–Cbtx V_H_H C2 with the C_H_2 and C_H_3 domains of a human IgG1 (V_H_H2-Fc antibody; Mr ∼80 kDa). Because of its Fc region and higher molecular weight, this antibody should offer prolonged serum persistence.

Our engineered V_H_H2-Fc antibody construct was successfully expressed *in planta* with an average yield of 129.50 mg kg^−1^ of fresh leaf tissue. Unfortunately, immunoblotting and N-terminal sequencing revealed that in addition to the correct V_H_H2-Fc antibody there was also a product consisting of the Fc region only. Since the ELISA used to quantitate the expression level detected all material with an Fc region, the average yield is likely closer to 50 mg kg^−1^ for the intact V_H_H2-Fc antibody, based on the densitometry results. SPR analysis showed that our plant produced V_H_H2-Fc antibody retains high affinity binding to α–Cbtx with a dissociation rate constant of 5.0×10^−5^ s^−1^. Our V_H_H2-Fc antibody also has potent *in vivo* neutralizing capacity against α–Cbtx comparable to the parent V_H_H C2 antibody.

Since the llama was immunized with crude *N. kaouthia* venom, the V_H_H library could also be panned against other components of the *N. kaouthia* venom. It would also be particularly valuable to select V_H_Hs against locally acting toxins including phospholipases A_2_ and metalloproteinases since these cause much of the tissue damage associated with Thai cobra snake bites [51]. Furthermore, methodologies should be developed to simultaneously select V_H_Hs against crude venom, while ensuring that all venom components are represented and neutralized. Mixtures of V_H_H C2 with current antivenom derived from horse should also be tested to determine if efficacy of the antivenom can be improved in terms of reducing its required dose. However, to reduce the tissue damage of whole venom at the site of a snake bite, V_H_Hs that bind components of the venom (discussed above) must also be produced before a solely V_H_H-based venom may prove effective.

In summary, we isolated high affinity llama single domain antibody fragments (V_H_Hs) against α-Cbtx from an immune library. The highest affinity binders, C2 and C20, were able to completely neutralize the lethal effects of α–Cbtx below 1x antibody:toxin molar ratios. For therapeutic efficacy, our results show that monomers should have a K_D_ of ca. 1 nM or better, thus requiring the creation of an immune library. Furthermore, we demonstrated that our plant-expressed V_H_H2-Fc antibody retained high affinity and also offered full protection to mice against the lethal effects of α–Cbtx. Further research towards the development of an antivenom therapeutic involving these anti-α-Cbtx V_H_Hs and V_H_H2-Fc antibody molecules should involve testing them as a combination, to determine whether they maintain tissue penetration capability and low immunogenicity, and whether they exhibit improved serum persistence and therapeutic efficacy.

## Supporting Information

Figure S1 **Final antiserum titre.** Llama serum from the final bleed (Day 134; solid line) and pre-immune serum (Dat -7; dash line) were serially diluted to determine the llama’s IgG titre to *N. kaouthia* venom using an end-point titration ELISA. The post-immune serum dilution which corresponded to three times the value of the background (Pre-immune sera) was determined to be 3.0×10^5^. Numbers are the average of triplicates. SEMs are shown with bars; when bars are not shown they are smaller than the symbol.
(TIF)

Figure S2 **Immunoblot of purified V_H_Hs under reducing conditions.** Immunoblots were probed with anti-penta His mouse mAb and goat-anti-mouse mAb conjugated to alkaline phosphatase. Lane 1, protein molecular weight standard; Lanes 2, V_H_H C2; Lane 3, V_H_H C19; Lane 4, V_H_H C20; Lane 5, V_H_H C43.
(TIF)

Figure S3 **Expression analysis of V_H_H2-Fc antibody using whole-plant infiltrations.** Coomassie-stained SDS-PAGE (**A**) and immunoblot (**B**) of plant total soluble protein (TSP) under non-reducing conditions. Immunoblots were probed with protein A conjugated to HRP. Lane 1, protein molecular weight standard; Lanes 2–6, 10 µg HCAb control series involving TSP from untreated plants spiked with purified llama HCAb (1200 ng, 600 ng, 300 ng, 150 ng and 75 ng, respectively); Lane 7, 10 µg TSP from untreated plants (negative control); Lane 8, 10 µg TSP from pooled V_H_H2-Fc antibody plants. See text for an explanation of asterisk.
(TIF)
