# Supplementary figures and images for: In Vivo Neutralization of α-Cobratoxin with High-Affinity Llama Single-Domain Antibodies (VHHs) and a VHH-Fc Antibody
Source: PLoS One. 2013 Jul 22;8(7):e69495. doi: 10.1371/journal.pone.0069495 (PMC3718736; doi:10.1371/journal.pone.0069495)

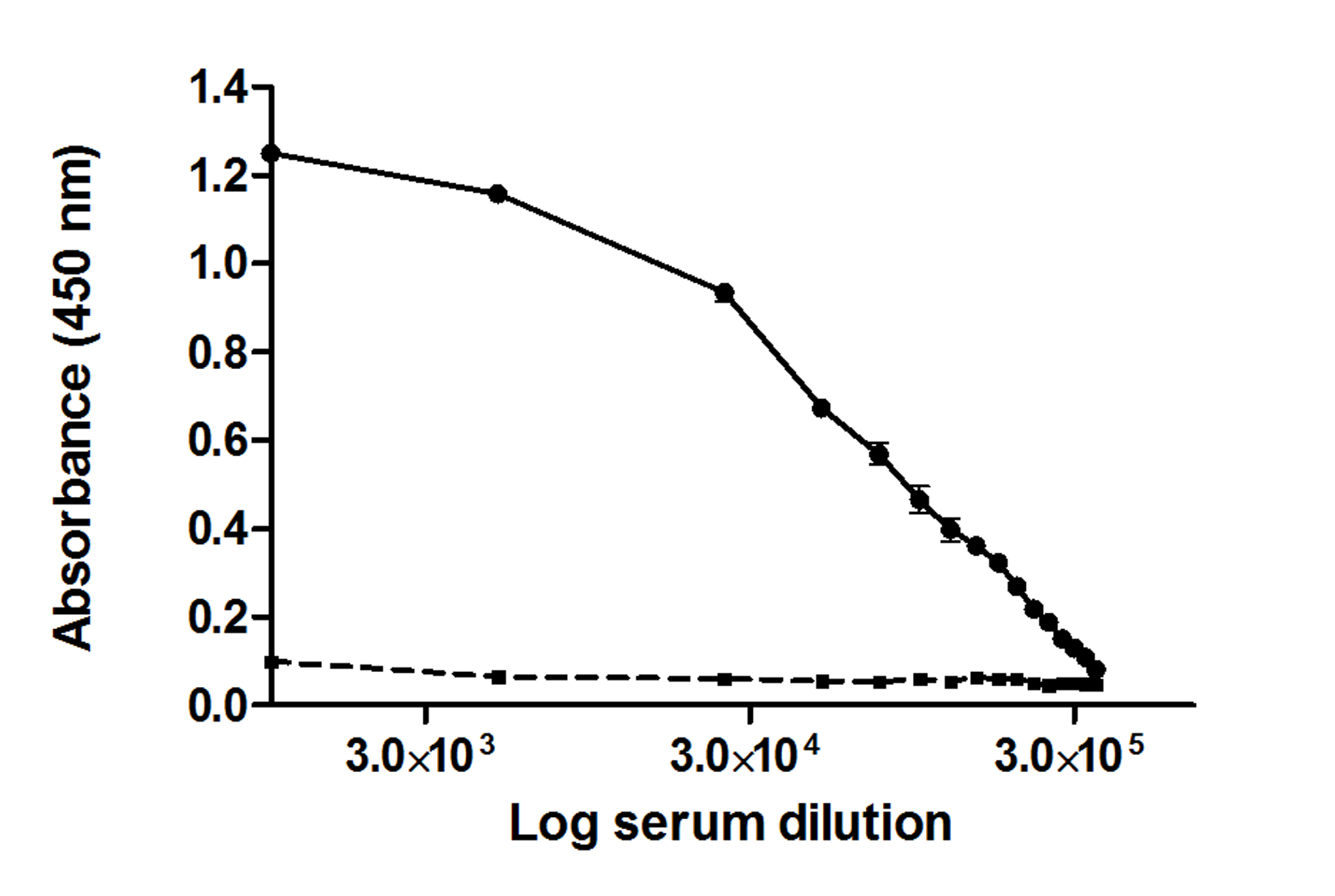

Supplement: Figure S1 — Final antiserum titre. Llama serum from the final bleed (Day 134; solid line) and pre-immune serum (Dat -7; dash line) were serially diluted to determine the llama’s IgG titre to N. kaouthia venom using an end-point titration ELISA. The post-immune serum dilution which corresponded to three times the value of the background (Pre-immune sera) was determined to be 3.0×10^5^. Numbers are the average of triplicates. SEMs are shown with bars; when bars are not shown they are smaller than the symbol. (TIF) [file pone.0069495.s001.tif]

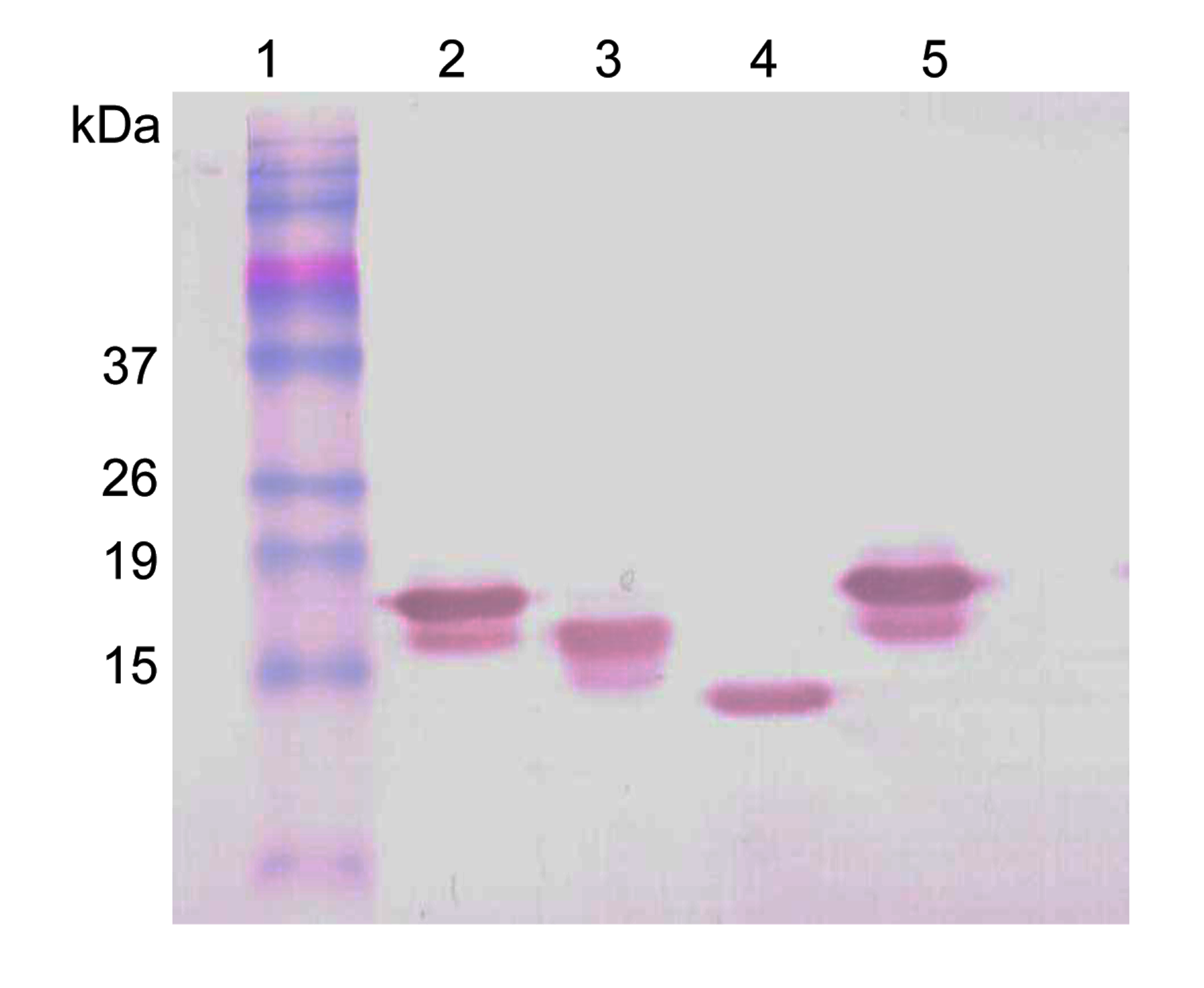

Supplement: Figure S2 — Immunoblot of purified VHHs under reducing conditions. Immunoblots were probed with anti-penta His mouse mAb and goat-anti-mouse mAb conjugated to alkaline phosphatase. Lane 1, protein molecular weight standard; Lanes 2, VHH C2; Lane 3, VHH C19; Lane 4, VHH C20; Lane 5, VHH C43. (TIF) [file pone.0069495.s002.tif]

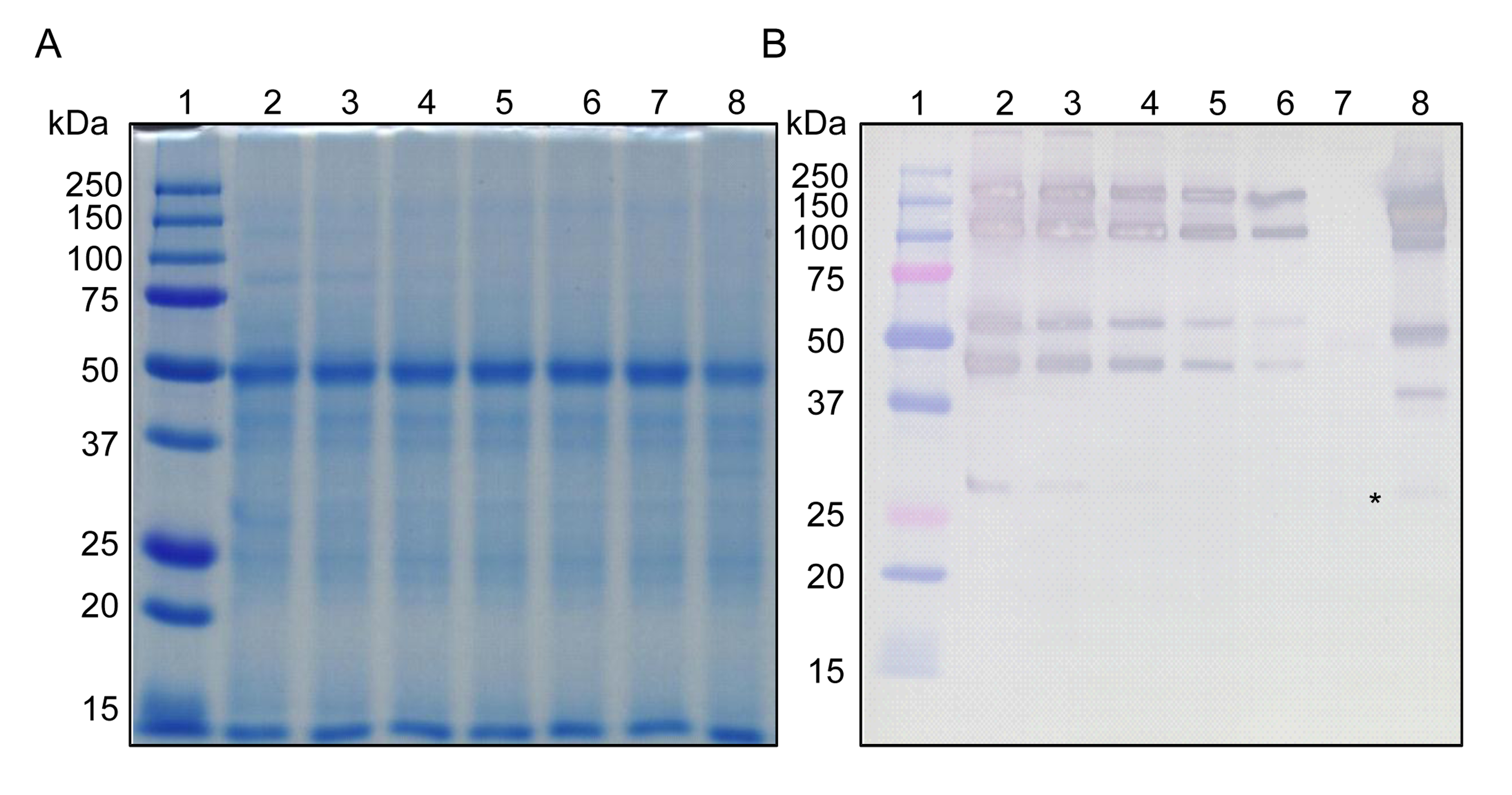

Supplement: Figure S3 — Expression analysis of VHH2-Fc antibody using whole-plant infiltrations. Coomassie-stained SDS-PAGE (A) and immunoblot (B) of plant total soluble protein (TSP) under non-reducing conditions. Immunoblots were probed with protein A conjugated to HRP. Lane 1, protein molecular weight standard; Lanes 2–6, 10 µg HCAb control series involving TSP from untreated plants spiked with purified llama HCAb (1200 ng, 600 ng, 300 ng, 150 ng and 75 ng, respectively); Lane 7, 10 µg TSP from untreated plants (negative control); Lane 8, 10 µg TSP from pooled VHH2-Fc antibody plants. See text for an explanation of asterisk. (TIF) [file pone.0069495.s003.tif]
